# Report from the World Health Organization's immunization and vaccines-related implementation research advisory committee (IVIR-AC) meeting, virtual gathering, 17–21 February 2025

**DOI:** 10.1016/j.vaccine.2025.127384

**Published:** 2025-08-13

**Authors:** Philipp Lambach, Sheetal Silal, Alyssa N. Sbarra, Mitsuki Koh, Rakesh Aggarwal, Habib Hasan Farooqui, Stefan Flasche, Alexandra B. Hogan, Sun-Young Kim, Kathy Leung, William J. Moss, Allison Portnoy, Meru Sheel, Xuan-Yi Wang

**Affiliations:** a Immunizations, Vaccines and Biologicals, World Health Organization, Geneva, Switzerland; b Modelling and Simulation Hub, Africa, University of Cape Town, Cape Town, South Africa; c Centre for Global Health, Nuffield Department of Medicine, Oxford University, Oxford, United Kingdom; d Johns Hopkins Bloomberg School of Public Health, Baltimore, MD, United States; e Sanjay Gandhi Postgraduate Institute of Medical Sciences, Lucknow, India; f College of Medicine, QU Health, Qatar University, Qatar; g Centre for Global Health, Charite, Berlin, Germany; h School of Population Health, Faculty of Medicine and Health, UNSW, Sydney, Australia; i Seoul National University, Seoul, South Korea; j School of Public Health, The University of Hong Kong, Hong Kong, SAR, China; k Department of Global Health, Boston University School of Public Health, Boston, United States; l Center for Health Decision Science, Harvard T.H. Chan School of Public Health, Boston, United States; m School of Public Health, Faculty of Medicine and Health, University of Sydney, Sydney, Australia; n Sydney Infectious Diseases Institute, Faculty of Medicine and Health, University of Sydney, Sydney, Australia; o Shanghai Institute of Infectious Disease and Biosecurity, Fudan University, Shanghai, China

**Keywords:** Vaccination, Immunization, IVIR-AC

## Abstract

The Immunization and Vaccines-related Implementation Research Advisory Committee (IVIR-AC) serves as the World Health Organization's (WHO) key advisory body for independently reviewing research that assesses the impact and value of vaccines, particularly using transmission and economic modeling analyses. During its first semi-annual meeting of 2025, held on 17–21 February and complemented by ad hoc sessions on 5 February, 11 April and 14 April, IVIR-AC provided feedback and recommendations across seven key sessions. This report summarizes the discussions and outcomes of the meeting. Topics covered included immunization research priorities in the WHO Eastern Mediterranean region, multi-model comparisons of typhoid conjugate vaccine schedules, a malaria intervention multi-model comparison, a full value assessment of invasive non-typhoidal *Salmonella* (iNTS) vaccination, an evaluation of improved influenza vaccines, vaccine impact modeling under the Immunization Agenda 2030 (IA2030) framework, and combination vaccines value assessment.

## 1 Context

Policies for vaccination programs should be guided by robust evidence. As an advisory body to the World Health Organization (WHO), the Immunization and Vaccines-related Implementation Research Advisory Committee (IVIR-AC) evaluates and reviews priority topics for the Strategic Advisory Group of Experts on Immunization (SAGE), the Product Development for Vaccines Advisory Committee (PDVAC), and the Immunization, Vaccines, and Biologicals (IVB) Department. These topics include vaccine effectiveness and impact studies, value assessments, and modeling analyses. While IVIR-AC does not have executive or decision-making authority [1], its primary role is to provide expert advice and recommendations to WHO through topic-based sessions presented during its semi-annual meetings. This report summarizes the discussions and recommendations from IVIR-AC's virtual meeting held on 5 February, 17–21 February, 11 April and 14 April 2025 [2].

## 2 Scope and objectives of meeting

The WHO's IVB Department and SAGE requested seven dedicated meeting sessions to address key topics, including:

• Immunization research priorities in the WHO Eastern Mediterranean Region (EMR)
• Multi-model comparisons for typhoid conjugate vaccine schedules
• Malaria intervention multi-model comparisons
• Full value assessment of invasive non-typhoidal *Salmonella* (iNTS) vaccination
• Full value of improved influenza vaccine assessment
• Vaccine impact modeling under the Immunization Agenda 2030 (IA2030) framework
• Combination vaccines value assessment

This report summarizes the objectives, discussions, and recommendations from each session.

## 3 Summary of sessions

Session 1: Overview of Immunization Research Priorities in WHO Eastern Mediterranean region.

IVIR-AC continues to take steps to improve linkages with and supporting WHO regional offices and Regional Immunization Technical Advisory Groups (RITAGs). The EMR comprises 22 member states, each with strong immunization programs, but faces challenges related to mobile and displaced populations and conflict. To foster engagement, identify regional research priorities, and explore areas of collaboration, IVIR-AC members and observers participated in a joint session with RITAG and National Immunization Technical Advisory Group (NITAG) members during the EMR-RITAG meeting.

IVIR-AC was asked to:

• Facilitate discussion on potential opportunities for connection between modelers within the EMR
• Comment on lessons from other regions that could benefit modelers within the EMR member states

During the session, IVIR-AC provided an overview of its scope and objectives to RITAG members. Presentations from regional experts highlighted ongoing research activities and challenges related to modeling and implementation across multiple topic areas and settings.

The EMR-RITAG plays a crucial role in guiding immunization policies and strategies within the region. During the session, the RITAG chair provided an overview of the group's composition and objectives, emphasizing its advisory role to the WHO EMR Regional Director on vaccine research and immunization strategies. Key functions of the RITAG include reviewing epidemiological data on vaccine-preventable diseases (VPDs), setting regional immunization goals in alignment with IA2030, and recommending strategies for National Immunization Programs, for consideration by National Immunization Technical Advisory Groups (NITAGs).

Saudi Arabia's Ministry of National Guard Health Authority (MNGHA), presented its vaccine implementation research portfolio, highlighting efforts in adolescent and adult immunization programs, particularly for human papillomavirus (HPV), influenza, and SARS-CoV-2 vaccines. Next, the American University of Beirut's WHO Collaborating Center for Bacterial Pathogens outlined its priorities in clinical, basic, and epidemiological sciences to inform public health policy. The Institut Pasteur Tunisia then presented key initiatives aimed at strengthening immunization research and capacity-building in the region. Notably, the institute highlighted the African Modeling and Analytics Academy for Women and its contributions to the Vaccine Impact Modelling Consortium (VIMC). Demonstrating the impact of modeling on immunization decision-making, the team shared an analysis of the cost-effectiveness of HPV vaccination and cervical cancer screening in Tunisia [3,4]. Then, a representative from the WHO EMR office provided an overview of the region's immunization priorities and challenges. Key issues included the need to vaccinate zero-dose children, especially in fragile, conflict-affected and vulnerable settings, support data-driven vaccine introduction decisions, and improve preparedness for VPD outbreaks through forecasting and risk assessments. Additionally, the WHO EMR Science, Information, and Dissemination team emphasized efforts to strengthen evidence generation and innovation to advance Sustainable Development Goals (SDGs), including increased support for clinical trials and immunization research.

Overall, IVIR-AC recognizes that EMR member states possess the epidemiological and modeling expertise relevant to vaccine-preventable diseases. IVIR-AC agrees with regional stakeholders that establishing joint working groups across the region, involving a diverse set of experts including local modelers, could facilitate implementation research collaboration, including establishing ecosystems and addressing shared challenges. IVIR-AC suggests that WHO provide further technical support for regional implementation research in key IA2030 priority areas, including cost-effectiveness and budget impact analysis, risk assessments, alternative vaccine schedule optimization, and social and behavioral research on vaccine hesitancy. IVIR-AC recommends establishing a regional database of vaccine researchers, particularly those with expertise in modeling and implementation science, to foster collaboration and knowledge sharing. IVIR-AC recommends establishing a mechanism to share information on research needs, available data, and modeling capacity in the region to facilitate the uptake of modeling to support immunization decision-making. Finally, IVIR-AC recommends identifying long-term funding opportunities to sustain modeling activities to support immunization programs.

Session 2: WHO Multi-Model Comparisons for Typhoid Conjugate Vaccine Adequate Schedules (MMC-TAS).

Typhoid fever remains an important public health problem in many low- and middle-income countries (LMICs), with an estimated 11 to 21 million cases annually [5]. The 2018 WHO position paper on typhoid vaccination [6] recommends a single dose of typhoid conjugate vaccine (TCV) in children aged 6 months and older in typhoid endemic regions. However, recent evidence suggests potential waning of effectiveness four to five years after vaccination among children vaccinated below the age of two years in high transmission settings. A SAGE working group [7] was formed to analyze this new evidence and to determine whether vaccine recommendations need to be updated. To support this evaluation, WHO commissioned multi-model comparisons across independent modeling teams to assess the optimal dosing schedule and the potential need for a booster dose. These models incorporate age-specific incidence data, waning immunity dynamics, and implementation and cost-effectiveness considerations and will be presented to the SAGE working group in May 2025.

IVIR-AC reviewed the modeling protocol and assumptions presented by modeling teams from Yale University, Stanford University, the Institute for Disease Modeling, and Burnet Institute. The models, which differ structurally, share common assumptions regarding health states, infection probabilities, vaccination, and waning immunity. IVIR-AC commends the methodological and systematic approach to model comparisons, including model validation against trial findings and exploring scenarios that capture routine and booster vaccination at different coverage levels and assumptions about the durability of vaccine efficacy. To facilitate understanding of this comprehensive work, IVIR-AC recommends that a summary be drafted to inform stakeholders to the objectives and outcomes of each model in a user-friendly format.

IVIR-AC also reviewed preliminary results presented by the four modeling teams, including incidence over time and cases averted, across many permutations of waning, incidence, and vaccination strategies. Results from probabilistic sensitivity analyses (PSA) from multiple groups were also presented. While sources of uncertainty vary across modeling teams, resulting uncertainty intervals across outputs were substantial across scenarios. IVIR-AC highlights the need for PSA comparing slow versus fast waning of TCV effectiveness across very high-, high-, and medium-burden settings. IVIR-AC emphasizes the importance for the modeling groups to explain key differences in their outputs relative to other models in the multi-model comparison. IVIR-AC recognizes that substantial uncertainty and wide prediction intervals in the presented PSA results preclude definitive conclusions about optimal vaccination strategies. The analysis should focus on identifying key uncertainty drivers, including vaccine waning rates, durability, force of infection, and cost variations, and use frontier curves and tornado diagrams for comparisons. Targeted investigation of these factors can help narrow prediction intervals, reduce ambiguity, and facilitate clearer comparisons between schedule options. Additionally, IVIR-AC recommends that the modeling groups using individual-based models conduct additional analyses to evaluate how individual variability in vaccine efficacy contributes to overall uncertainty in their model estimates and predictions.

The Yale team presented preliminary cost-effectiveness findings, suggesting different preferred vaccination strategies across regions (i.e., levels of antimicrobial resistance), waning of effectiveness scenarios, burden settings (i.e., medium, high, very high), and willingness to pay per disability-adjusted life year (DALY) averted. In slow waning scenarios, populations with a lower willingness to pay favored routine vaccination at 9 months with a catch-up campaign vaccination strategy and those with higher willingness to pay preferred boosters at 5 and 10 years with a catch-up campaign vaccination strategy. In fast waning scenarios, most combinations exhibited a preference for a vaccination strategy with boosters at 5 and 10 years with a catch-up campaign. IVIR-AC recommends assessing the key drivers of differences between optimal routine vaccination schedules (e.g., 9 months vs. 5 years, with or without boosters) and evaluating their cost-effectiveness in a competing choice framework in relation to the force of infection in each setting. Additionally, systematic exploration of uncertainties—particularly around vaccination coverage differences between age-based strategies—should be prioritized.

Given the substantial uncertainty in model outputs, IVIR-AC suggests the need for a better assessment of the robustness of various vaccination strategies by quantifying how frequently the simulations meet predefined effectiveness thresholds (e.g., percentage of simulations that achieve a specified reduction in incidence over a specified time) and cost-effectiveness thresholds (e.g., cost-effectiveness acceptability curves). Additionally, given substantial uncertainty surrounding durability of the protection provided by the vaccine and effectiveness of a booster dose, IVIR-AC advises careful consideration in communicating these model results and using these for policy decisions until further evidence is available. IVIR-AC notes that re-evaluation of model scenarios may be necessary as new data emerge. Finally, IVIR-AC suggests that future modeling work may explore surveillance strategies to allow reactive booster dose implementation based on emerging evidence on durability of protection provided by vaccination in the early years of life.

Session 3: Malaria multi-model comparison of prioritized interventions (M3CPI).

Scientific advancements have improved our understanding of transmission dynamics and led to the development of new insecticides and vaccines against *Plasmodium falciparum* malaria. However, budget constraints necessitate careful prioritization of malaria interventions. The WHO Global Malaria Programme and Regional Offices released the “Guiding principles for prioritizing malaria interventions in resource-constrained country contexts to achieve maximum impact” (GPP) [8] to guide decision-making on efforts to achieve maximum impact of national malaria control programs. A new project will use two mathematical models to inform updates to the GPP document by modeling the impact and cost-effectiveness of different malaria control interventions in combination rather than individually.

IVIR-AC was asked to provide feedback on the robustness of the protocol for multi-model comparison analyses and to discuss the next steps to finalize results that will be presented to IVIR-AC in September 2025.

The session included presentation by a WHO team on considerations for an M3CPI costing analysis, emphasizing core cost-effectiveness principles and standardization of modeling approaches. Overall, IVIR-AC acknowledges the robustness of the multi-model comparative analysis protocol and supports the preparatory efforts leading up to the presentation of results at the IVIR-AC meeting in September 2025. IVIR-AC also acknowledges the ambitious scope of this project and the tight timeline for completion. Modeling teams will use incremental cost per disability-adjusted life year (DALY) averted as the outcome measure and conduct sensitivity analyses. Additionally, a project consultant highlighted the need for key shared assumptions while maintaining model independence. Three main archetypes have been selected to capture variations in malaria epidemiology, ecology, and demography. IVIR-AC notes that given the complexities of malaria transmission within and across archetypes, harmonizing the models remains a significant challenge. Modeling teams must also consider the indirect impact on mortality and age-specific mortality, as well as assess intervention impact based on implementation timing and target population. IVIR-AC recommends capturing and communicating uncertainty due to the lack of empirical data to fully explore the impact of various interventions and their combinations. IVIR-AC also recommends prioritizing combinations of malaria interventions with the most programmatic relevance, ensuring that modeling analyses align with programmatic needs. Finally, IVIR-AC emphasizes the importance of in-depth stakeholder engagement to determine the appropriate prioritization of interventions for the modeling analyses.

*Session 4*: Invasive non-typhoidal *Salmonella* (iNTS) Full Value of Vaccine Assessment.

Invasive non-typhoidal *Salmonella* (iNTS) poses a significant health burden, particularly among children in sub-Saharan Africa, with antimicrobial resistance complicating treatment. As developing iNTS vaccines requires investment from multiple stakeholders, the Full Value of Vaccine Assessment (FVVA) was developed to assess the business case, investment case, and broader societal benefits of iNTS vaccines. IVIR-AC reviewed the FVVA for clarity and completeness.

The business case, presented by Shift Health, assessed market potential and return on investment for a trivalent vaccine with one- and two-dose regimens. Demand forecasts estimated 405 million and 834 million cumulative routine doses by year 12 of the immunization program, with projected revenues of US$1.62 billion (one-dose) and US$2.69 billion (two-dose). Profitability was achieved only at a price/cost margin of US$3.00 per dose. For the business case, IVIR-AC recommends considering an additional scenario where iNTS vaccine is not initially prioritized for Gavi funding in Gavi-eligible countries. IVIR-AC also recommends that vaccine impact projections should account for scenarios where routine vaccine coverage falls lower than that achieved by the third dose of diphtheria-tetanus-pertussis vaccine (DTP3) and the first dose of measles-containing vaccine (MCV1), or where catch-up campaign coverage is lower than currently assumed.

The International Vaccine Institute (IVI) presented the investment case, including a systematic review of 38 studies on iNTS economic burden. A stakeholder survey across eight African countries indicated a preference for a trivalent vaccine over a bivalent vaccine, depending on price and efficacy. Further, a bivalent vaccine was estimated to be cost-effective in sub-Saharan Africa, while a trivalent vaccine was estimated to be cost-effective in both sub-Saharan Africa and South-East Asia.

NYU/LSHTM presented an analysis of the broader societal impacts of iNTS vaccination [9], including healthcare costs, productivity loss, and household insecurity. Preliminary findings suggest that iNTS vaccination would have the greatest impact and cost-effectiveness among lower socio-economic groups. IVIR-AC recommends that equity analyses integrate diverse and country-specific data and explore equity indicators, such as maternal education and other risk factors that influence iNTS uptake.

Overall, IVIR-AC commends the comprehensive approach undertaken in the conceptualization of the components of the FVVA for iNTS vaccines that were presented and supports the selected modeling framework for evaluating evidence to support decision-making across vaccine development and implementation, considering sustainable socio-economic and public health impacts. IVIR-AC acknowledges the complexity of evidence being generated by different partners across the business case, investment case, and broader societal impact analysis, and emphasizes the importance of synthesizing findings to ensure a cohesive FVVA. IVIR-AC recommends presenting results separately for bivalent iNTS vaccine and for trivalent vaccine (i.e., bivalent iNTS + TCV) to facilitate an assessment of the incremental benefits that a trivalent iNTS vaccine would have in settings where a TCV vaccine has already been introduced.

Generally, IVIR-AC acknowledges a lack of primary data, particularly on the economic burden of iNTS in LMICs, and suggests that future studies generate these data to strengthen modeling and FVVA findings. When disseminating FVVA results, IVIR-AC recommends addressing findings in the multi-stakeholder analyses in relation to lack of awareness and community acceptance. Finally, IVIR-AC also recommends contextualizing the messaging regarding the long-term and broader benefits of vaccines compared to other interventions for reducing iNTS burden.

As the FVVA will be put together in the upcoming months, IVIR-AC advises a review of the final analysis and results and to discuss the dissemination plan and optimal use of the FVVA among end users as the FVVA comes to completion.

Session 5: Full value of improved influenza vaccine assessment (FVIVA).

Influenza remains a major global health burden, yet seasonal vaccines are underutilized [10,11], with only 15 % of LMICs having national influenza vaccine programs [12]. Barriers to program implementation include high costs, limited access to WHO-recommended vaccines, and limited awareness of benefits of current vaccines [13]. Since 2023, WHO has been developing a full value of improved influenza vaccines assessment (FVIVA) to assess the value of existing vaccines and the potential impact of future “improved vaccines.” [14] FVIVA integrates multiple workstreams to define vaccine characteristics and estimate their health and economic impact. IVIR-AC was invited to provide feedback on the key conclusions from the analyses contributing to the FVIVA and priorities for future research.

The project team presented an integrated analysis covering impact, cost-effectiveness, and economic surplus. Vaccine scenarios included current seasonal vaccines (baseline), minimally improved efficacy/duration vaccines, significantly improved efficacy and duration vaccines, significantly improved breadth and duration vaccines, and a ‘game changer’ vaccine with enhanced efficacy, breadth, and duration.

A compartmental transmission model was applied across epidemic periods in exemplar countries representing different influenza transmission zones. Estimated health outcomes included infections, hospitalizations, deaths, and DALYs. Economic evaluation incorporated willingness-to-pay thresholds, delivery costs, and treatment expenses to determine country-specific vaccine threshold prices and global net monetary benefits. IVIR-AC commends the various efforts to address its previous recommendations [14,15], noting that the presented workstream addresses relevant points that were suggested for consideration. Given the country-level analyses [[16], [17], [18]] as a foundation for model calibration, IVIR-AC supports the global analysis, which extrapolates the modeling framework to assign ‘influenza transmission zones’ (ITZs) based on exemplar countries. IVIR-AC notes that, given the parameterization of the analyses, conclusions will be limited to archetypes with characteristics of ITZs but could indicate where market demand for improved influenza vaccines is likely to be highest.

Preliminary results indicated that all improved vaccines would substantially reduce influenza mortality, particularly in Europe, the Americas, and the Western Pacific. ‘Significantly improved’ vaccines and ‘game changer’ vaccines showed similar estimated impact, both outperforming minimally improved vaccines. Minimally improved duration vaccines were estimated to be more effective than minimally improved efficacy vaccines. Current seasonal vaccines were estimated to require vaccinating three people to prevent one influenza virus infection, on average, whereas ‘game changer’ vaccines were estimated to prevent over two infections per vaccinated individual. Under the forecasted demand, cost-effectiveness varied by income level; in high-income countries, improved vaccines could be viable even at US$100+ per dose; comparatively, in low-income settings, incremental cost-effectiveness ratios were lower in magnitude. Net monetary benefit analyses highlighted price-dependent feasibility across different regions. As characteristics of candidate vaccines become known, IVIR-AC acknowledges that the global incremental net monetary benefits results support an initial prioritization of improved duration over improved efficacy to achieve a globally cost-effective profile.

To fill evidence and methodological gaps, IVIR-AC suggests that future research incorporate additional analytic considerations in both a global analysis and country-level analyses for improved influenza vaccines, including:

• An evaluation of delivery and implementation strategies to targeted age groups and/or targeted high-risk (e.g., occupational) groups with both routine and campaign delivery modalities;
• An analytic framework that goes beyond the health system perspective to incorporate either a full or restricted societal perspective (i.e., patient non-medical costs and time/income loss);
• Framing scenarios as competing choices so that an optimal delivery strategy could be determined based on the specified assumptions; and
• A scenario that includes combined administration of both seasonal and universal vaccines to mitigate the risk of vaccine escape variants.

Additionally, given considerations of feasibility and affordability in LMICs, IVIR-AC recommends conducting additional budget impact analyses. Finally, as additional trial data become available, IVIR-AC suggests conducting updated analyses with enhanced analytic specificity.

Session 6: IA2030 vaccine impact modeling.

WHO IVB first estimated vaccine impact for the Impact Goal Indicator 1.1 (“number of future deaths averted through immunization”) of Immunization Agenda 2030 (IA2030) in 2021, projecting approximately 50 million future deaths averted if coverage targets are met by 2030 [19,20]. IVIR-AC has reviewed and supported previous updates of this work [21,22]. The current project aims to refine these estimates by incorporating additional diseases, morbidity impact (DALYs), and non-routine immunization activities. Challenges include consolidating divergent modeling methods, extrapolating from LMICs to high-income countries (HICs), and ensuring methodological transparency. IVIR-AC was asked to provide feedback on model methodology using measles transmission data, the optimal level of complexity, and potential additional validation exercises.

During the session, the IA2030 modeling team presented efforts to unify methodologies used in IA2030 and in analyses to mark the 50th anniversary of the Expanded Programme on Immunization (EPI@50), which differ in inputs, extrapolation methods, and definitions of vaccine impact (year-of-vaccination versus calendar year). Given different methodologies, purposes, audiences, and timeframes, IVIR-AC recommends adopting only relevant and appropriate methodologies from the EPI@50 exercise to improve IA2030 estimates going forward. IVIR-AC recommends that methodologic updates balance improved accuracy in estimates versus the practicality of interpretation. IVIR-AC recommends examination of the impact that changing methods will have on communication of methods and results to stakeholders.

Validation exercises using VIMC outputs, including measles impact estimates, found low root mean squared error and mean absolute error but moderate R^2^ (coefficient of determination), indicating output variability. IVIR-AC suggests that additional validation exercises could include subsetting VIMC results for diseases with delayed expected reductions in burden, such as HPV, and quantifying sensitivity to variation in impact estimates from both VIMC measles models. Further model selection via Akaike's Information Criterion (AIC) identified key predictors, including vaccine coverage, maternal mortality, and Gini coefficients, with stunting as an additional predictor for measles models. To reduce model overfitting and improve understanding of drivers of vaccine impact, IVIR-AC recommends limiting the suite of potential statistical models via expert-guided predictor selection. Additionally, IVIR-AC supports region- and income-specific model selection and suggests that it may be possible to reduce the total number of combinations analyzed.

Future steps include refining models by year-of-vaccination, simplifying frameworks for transparency, and incorporating the Sustainable Development Index (SDI) as a universal predictor. These refinements aim to enhance the clarity, usability, and accuracy of vaccine impact estimates across diverse epidemiological settings.  IVIR-AC supports the vaccine impact calculations using the year-of-vaccination method to better capture impact across different diseases and promote methodological consistency and comparability in future IA2030 updates. IVIR-AC supports exploring the use of SDI as a predictor in statistical models.

Session 7: Combination vaccines value assessment.

Vaccination programs have been very successful over the last 50 years [20]. In order to address the burden of vaccine-preventable diseases and deaths, the number of vaccines has increased, resulting in an increased number of immunization visits, several injections during single visits, logistical complexities, costs, and reduced acceptability. In addition, the promise of new vaccine development and introductions continues to add complexities across competing priorities [23]. Combination vaccines could be a solution to this mounting dilemma though improving vaccine delivery (e.g., timeliness, coverage), health impact (e.g., including equity considerations), administration (e.g., fewer syringes, cold chain space), and acceptance (e.g., greater demand). Further, there are likely to be additional advantages, such as simpler logistics and reduced generation of biomedical waste. However, important factors need to be considered, including programmatic challenges (e.g., vaccines proposed to be combined should have similar schedules and target populations), technical feasibility (e.g., different formulations or platforms), commercial uncertainty (e.g., unknown demand), clinical development and regulatory challenges, and policy vacuum. WHO and PATH have embarked on a new project to develop a framework that will identify new vaccine combinations that represent value for immunization programs and are programmatically compatible, technically feasible and commercially attractive. This project aims to inform investments in combination vaccine research and development and market shaping, and to inform policy and regulatory decisions about the use and introduction of combination vaccines.

During the session, the team from PATH presented a project overview, including timelines and engagement plans with other technical and advisory groups such as PDVAC, SAGE, and a technical advisory group. The framework developed will: (1) identify vaccines based on specific entry criteria; (2) combine vaccines for pairwise analyses; (3) identify combinations that are programmatically compatible; (4) identify combinations that are technically feasible; (5) consider commercial potential; and (6) conduct health economics value assessment and consider priorities. IVIR-AC supports the stepwise plan for identification of potentially useful combination vaccines and the consultative approach. IVIR-AC recommends early engagement and a systematic approach to seek feedback from key stakeholders (e.g., RITAGs, NITAGs, EPI managers).

Additionally, a value assessment will be conducted to identify, prioritize and use an enhanced list of metrics to better value combination vaccines and to increase information available to vaccine developers and other critical partners. For example, the benefit of fewer injections would decrease pain and anxiety while improving coverage and timeliness, and related metrics include increased coverage and willingness to pay to avoid pain and anxiety from stand-alone injections. Although it is reasonable to focus on the potential benefits of combination vaccines, IVIR-AC recommends also examining potential risks (e.g., reduced immunogenicity, increased procurement costs) and unintended consequences (e.g., perceptions regarding safety of one antigen may reduce uptake of the combination vaccine, reduced number of manufacturers of single-antigen vaccines). Finally, IVIR-AC recommends formalizing and refining the protocol to guide antigen selection by outlining specific exclusion criteria, including providing explicit definitions and descriptions of considerations, for consultation with key stakeholders.

## 4 Recurring themes

• *Communication and stakeholder engagement:* It is critical that communication is tailored to the needs of multiple stakeholders as seen in the discussion on country applications of TCV; translating archetype analyses to country implementation for malaria intervention prioritization; addressing stakeholders needs across the vaccine development pipeline for iNTS vaccination; early and systematic engagement with stakeholders on combination vaccines; and communicating methodological changes for IA2030 estimates, including engaging stakeholders to ensure that methodological concerns (e.g., considerations related to IA2030) are addressed comprehensively.

• *Exploring uncertainty:* The importance of exploring and communicating uncertainty where parameter estimates are based on limited evidence was emphasized, particularly in the context of waning protection of TCV and the impact of malaria interventions. This approach is critical to making informed policy decisions despite data limitations. Targeted investigation of these key drivers of uncertainty can help narrow prediction intervals, reduce ambiguity, and facilitate clearer comparisons between modelled scenarios. Relatedly, the need to systematically characterize and compare scenarios to assess incremental benefits was highlighted across multiple sessions.
• *Value of archetype analysis:* The use of archetype analysis was highlighted in several sessions, including on TCV, prioritizing malaria interventions, and the FVIVA. This method allows for the examination of diverse epidemiological, ecological, and demographic variations, and provides a robust framework for evaluating vaccine impact and cost-effectiveness.
• *Harmonization* versus *independence in multi-model comparisons:* The multi-model comparison analyses (i.e., TCV and malaria intervention prioritization) demonstrated that while harmonization of parameters, assumptions, and analysis ensures consistency and comparability, preserving model independence allows for diversity and a broader range of insights.

IVIR-AC will next convene for its bi-annual meeting from 2 to 5 September 2025.

## WHO author disclaimer

The authors have no interests to declare. Philipp Lambach and Mitsuki Koh work for the World Health Organization (WHO). The authors alone are responsible for the views expressed in this publication and they do not necessarily represent the decisions, policy or views of the WHO.

## CRediT authorship contribution statement

**Philipp Lambach:** Writing – review & editing, Supervision, Project administration, Conceptualization. **Sheetal Silal:** Writing – review & editing, Investigation, Conceptualization. **Alyssa N. Sbarra:** Writing – original draft, Conceptualization. **Mitsuki Koh:** Writing – review & editing, Project administration. **Rakesh Aggarwal:** Writing – review & editing, Investigation. **Habib Hasan Farooqui:** Writing – review & editing, Investigation. **Stefan Flasche:** Writing – review & editing, Investigation. **Alexandra B. Hogan:** Writing – review & editing, Investigation. **Sun-Young Kim:** Writing – review & editing, Investigation. **Kathy Leung:** Writing – review & editing, Investigation. **William J. Moss:** Writing – review & editing, Investigation. **Allison Portnoy:** Writing – review & editing, Investigation. **Meru Sheel:** Writing – review & editing, Investigation. **Xuan-Yi Wang:** Writing – review & editing, Investigation.

## Declaration of competing interest

The authors declare the following financial interests/personal relationships which may be considered as potential competing interests: P. L. is supported by the Gates Foundation. S. S. was supported by the World Health Organization for this work. A. N. S. was financially supported by the World Health Organization for this work, and is additionally supported by the Gates Foundation, Gavi, the Vaccine Alliance, and the National Institutes of Health. M. K. is supported by the Gates Foundation. A. B. H. was supported by the Australian National Health and Medical Research Council for this work, is additionally supported by PATH, the World Health Organization, and Gavi, the Vaccine Alliance, and has received consulting fees from the Australian NSW Ministry of Health, WHO Europe and Asian Development Bank. A. P. is supported by Gavi, the Vaccine Alliance, Imperial College London, the Gates Foundation, and the World Health Organization. A. N. S. and A. B. H. report travel related support from the World Health Organization to attend previous IVIR-AC meetings. All other authors have no declarations.

## Data Availability

No data was used for the research described in the article.

## Appendix A Supplementary data

Supplementary material Image 1
