# Supplementary material for: Report from the World Health Organization's immunization and vaccines-related implementation research advisory committee (IVIR-AC) meeting, virtual gathering, 17–21 February 2025
Source: Vaccine. 2025 Aug 13;61:None. doi: 10.1016/j.vaccine.2025.127384 (PMC12447087; doi:10.1016/j.vaccine.2025.127384)
Supplement: Supplementary file 1 — Supplementary material [file mmc1.docx]

Data Statement

There is no data contained within this report. For additional details per each session, please see: <https://www.who.int/news-room/events/detail/2025/02/17/default-calendar/immunization-and-vaccines-related-implementation-research-advisory-committee-(ivir-ac)---february-2025>.
